# Supplementary material for: Multilevel Regulation of NF‐κB Signaling by NSD2 Suppresses Kras‐Driven Pancreatic Tumorigenesis
Source: Adv Sci (Weinh). 2024 Jun 18;11(30):2309387. doi: 10.1002/advs.202309387 (PMC11321637; doi:10.1002/advs.202309387)
Supplement: Supplementary file 1 — Supporting Information [file ADVS-11-2309387-s001.pdf]

ADVANCED SCIENCE
Open Access

Supporting Information

for *Adv. Sci.*, DOI 10.1002/advs.202309387

Multilevel Regulation of NF-κB Signaling by NSD2 Suppresses *Kras*-Driven Pancreatic Tumorigenesis

Wenxin Feng, Ningning Niu, Ping Lu, Zhuo Chen, Hanyu Rao, Wei Zhang, Chunxiao Ma, Changwei Liu, Yue Xu, Wei-Qiang Gao, Jing Xue and Li Li**

Figure S1.

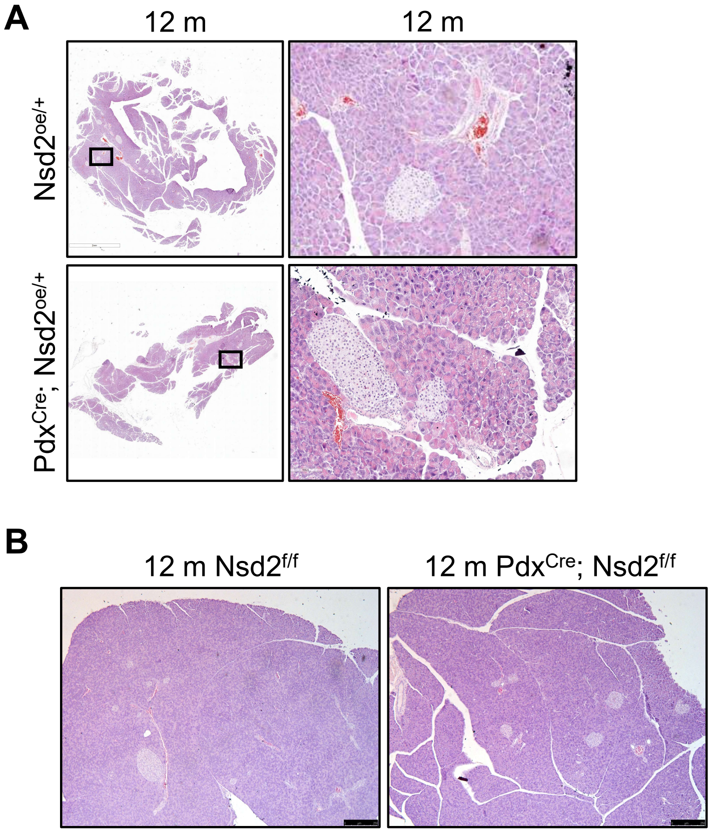

Fig. S1. Histological examination of *Nsd2*$^{OE/+}$, *Pdx*^{Cre}; *Nsd2*$^{OE/+}$, *Nsd2*$^{f/f}$ and *Pdx*^{Cre}; *Nsd2*$^{f/f}$ mice at 12 months of age.

(A) Pancreatic tissues from *Nsd2*$^{OE/+}$ and *Pdx*^{Cre}; *Nsd2*$^{OE/+}$ mice at 12 months of age for staining of HE (n = 6 per group). (B) Pancreatic tissues from *Nsd2*$^{f/f}$ and *Pdx*^{Cre}; *Nsd2*$^{f/f}$ mice at 12 months of age for staining of HE (n = 6 per group).

Figure S2.

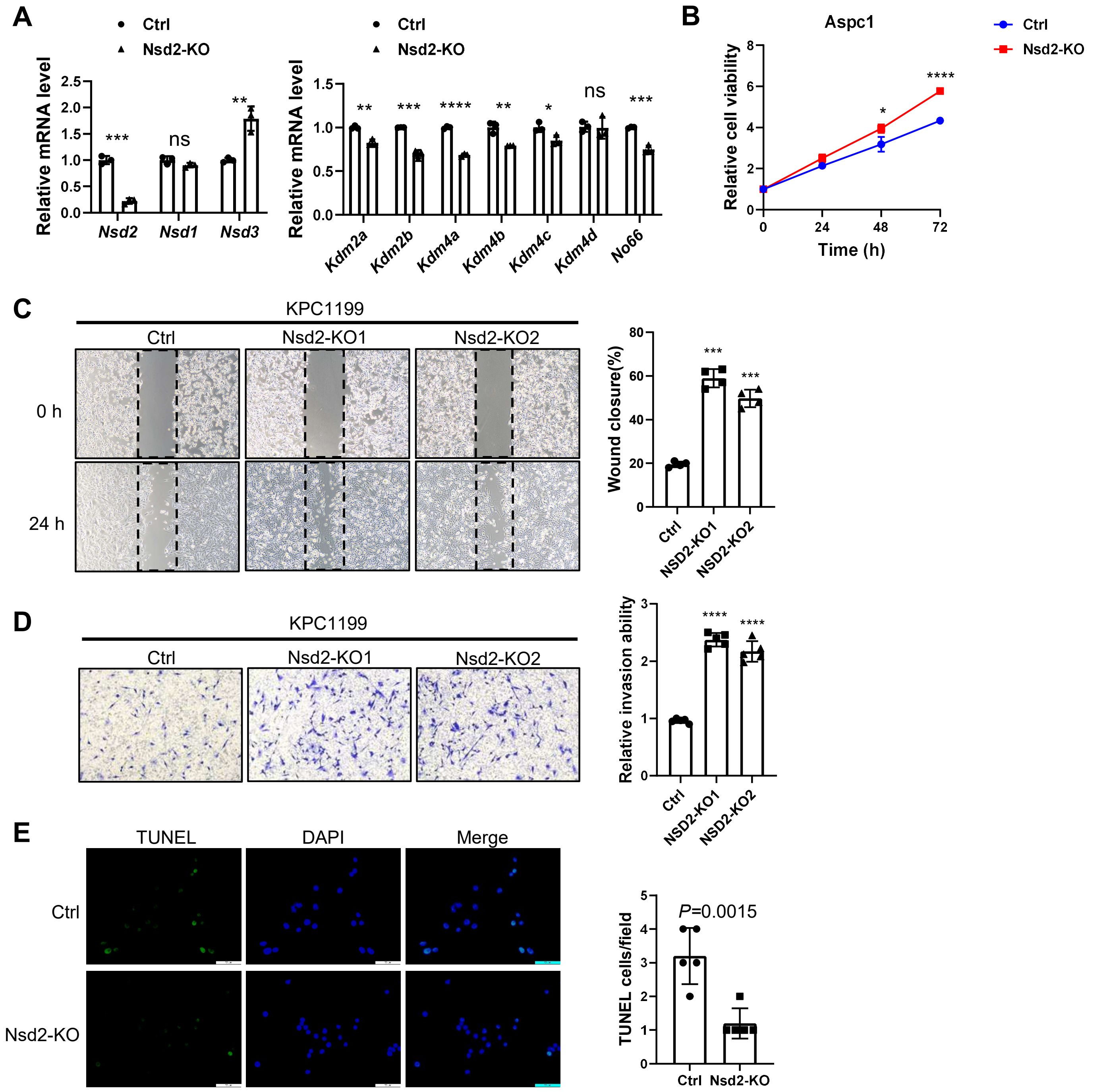

Fig. S2. ***Nsd2*** **deletion promotes cell migration and invasion.**

(A) RT-qPCR analysis of *Nsd2*, *Nsd1*, *Nsd3*, *Kdm2a*, *Kdm2b*, *Kdm4a*, *Kdm4b*, *Kdm4c*, *Kdm4d* and *No66* mRNA levels of Ctrl and Nsd2-KO derived from KPC1199 cells. Experiments were repeated at least three times, with similar results. (B) CCK8 assay of Ctrl and Nsd2-KO cells (Aspc1 derived). Experiments were repeated at least three times, with similar results. (C) Wound healing assay of Ctrl, Nsd2-KO1 and Nsd2-KO2 cells (KPC1199 derived) for 24 hours, and quantitation results are shown in the right (n = 5). Experiments were repeated at least three times, with similar results, and representative images are shown. (D) Transwell-based invasion assay of Ctrl, Nsd2-KO1 and Nsd2-KO2 cells (KPC1199 derived) for 24 hours, and quantitation results are shown in the right (n = 5). Experiments were repeated at least three times, with similar results, and representative images are shown. (E) TUNEL staining of Ctrl, Nsd2-KO cells (KPC1199 derived), and quantitation results are shown in the right (n = 5). Scale Bars: 50 μm. Experiments were repeated at least three times, with similar results, and representative images are shown.

Figure S3.

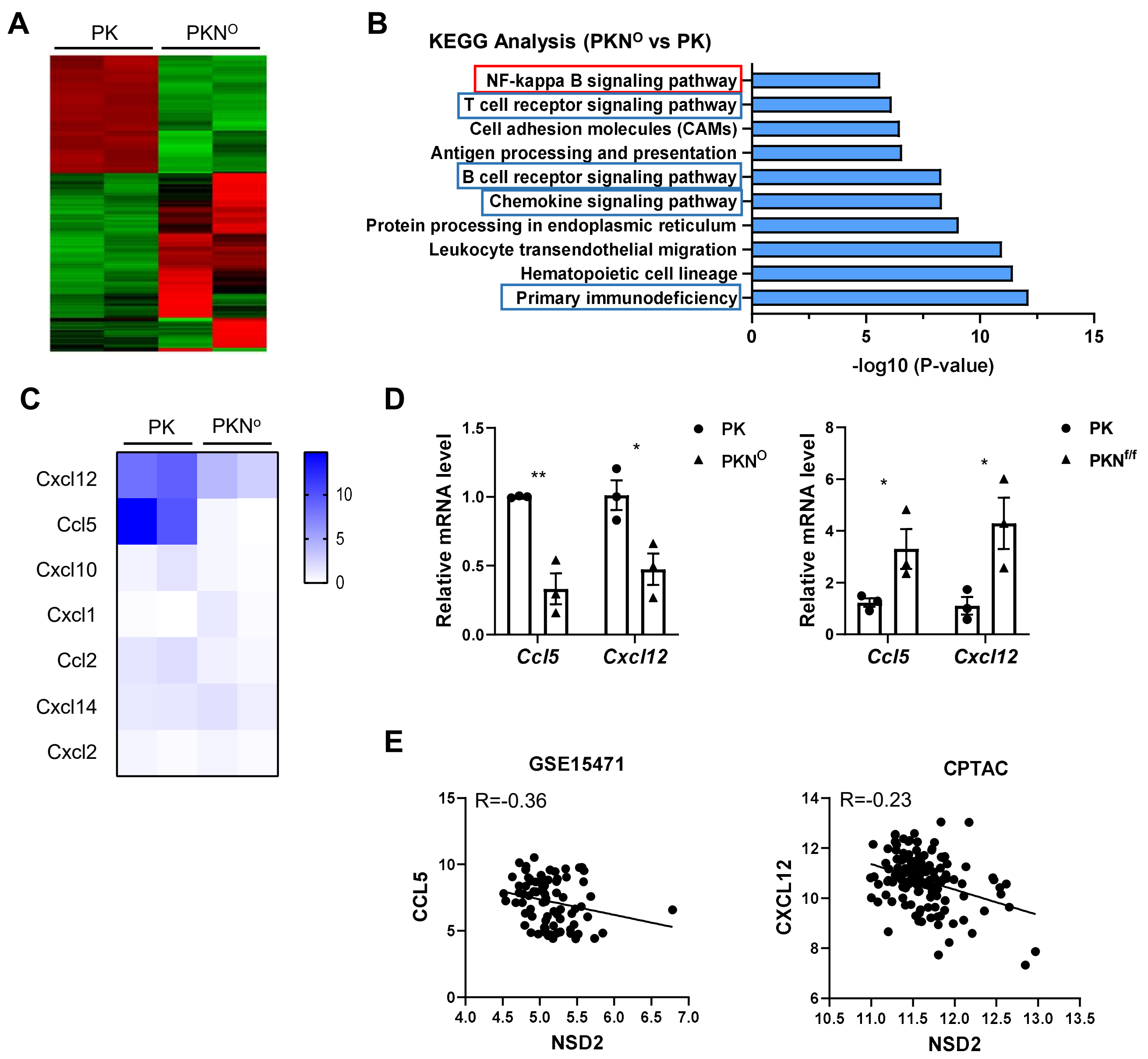

Fig. S3. **NSD2 overexpression inhibits NF-κB signaling pathway.**

(A) Heat map of RNA-seq data to compare the gene expression (PK and PKN^{O} mice). (B) KEGG analysis of gene expression changes in RNA-Seq data (PK and PKN^{O} mice). (C) Heatmap summarizes the RNA-seq results of gene expression related to Chemokine/NF-κB pathway interactions in PDAC. (D) RT-qPCR analysis of the relative mRNA levels of Cxcl5 and Cxcl12 of indicated mice. Experiments were repeated at least three times, with similar results, and representative images are shown. (E) The Pearson product-moment pair-wise gene correlation analysis between NSD2 and CCL5 or CXCL12 in GSE15471 and CPTAC databases.

Figure S4.

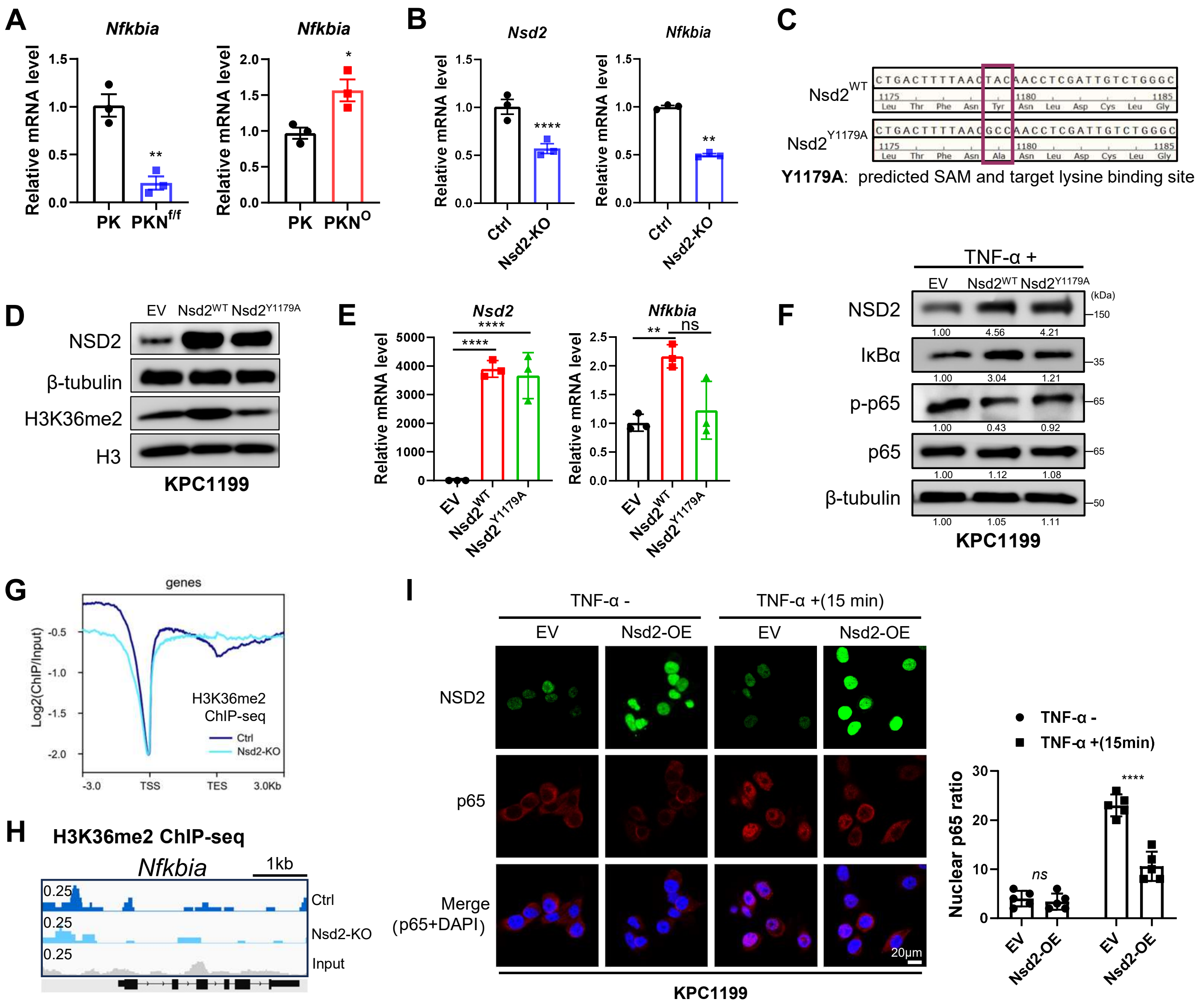

Fig. S4. NSD2-mediated H3K36me2 promotes Nfkbia expression.

(A) RT-qPCR analysis of Nfkbia mRNA level of PK and PKN$^{f/f}$ mice (left). RT-qPCR analysis of Nfkbia mRNA level of PK and PKN^{O} mice (right). Experiments were repeated at least three times, with similar results. (B) RT-qPCR analysis of Nsd2 and Nfkbia mRNA levels of Ctrl and Nsd2-KO cells (KPC1199 derived). Experiments were repeated at least three times, with similar results. (C) Schematic diagram showing mutation site of NSD2. (D) Western blotting analysis of NSD2 and H3K36me2 expressions in EV, Nsd2^{WT} and Nsd2^{Y1179A} cells (KPC1199 derived). Experiments were repeated at least three times, with similar results. (E) RT-qPCR analysis of Nsd2 and Nfkbia mRNA levels of EV, Nsd2^{WT} and Nsd2^{Y1179A} cells (KPC1199 derived). Experiments were repeated at least three times, with similar results. (F) Western blotting analysis of NSD2, IκBα, p-p65, p65 levels in EV, Nsd2^{WT} and Nsd2^{Y1179A} cells (KPC1199 derived). Experiments were repeated at least three times, with similar results. (G) Normalized read density of H3K36me2 ChIP-seq signals of Ctrl and Nsd2-KO cells (derived from PANC1) from 3 kb upstream of the TSS to 3 kb downstream of the TES. (H) Snapshot of H3K36me2 ChIP-Seq signals at the Nfkbia gene loci in PANC1 cells. (I) Immunofluorescence analysis of NSD2 and p65 expressions in EV and Nsd2-OE cells (KPC1199 derived) treated without or with TNF-α (20 ng/ml) for 15 min. Quantification of nuclear p65 mean intensity is shown at the right. Experiments were repeated at least three times, with similar results, and representative images are shown.

Figure S5.

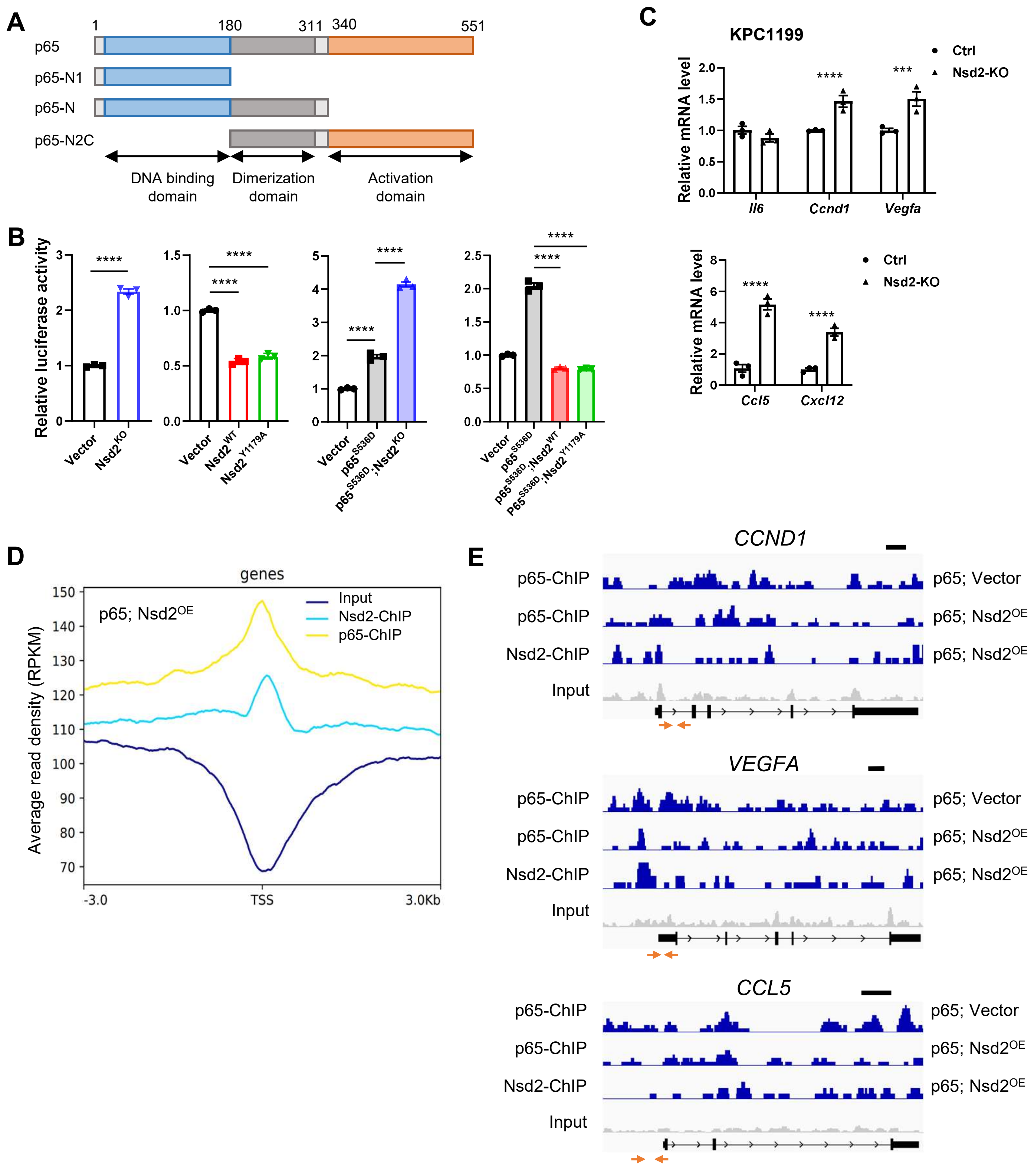

Fig. S5. **NSD2 inhibits the transcriptional activity of p65.**

(A) Diagram of the truncation mutants of p65. The N-terminal DNA binding domain, dimerization domain and the activation domain are shown by blue, gray and yellow boxes, respectively. (B) Luciferase assays of NF-κB activity in 293T cells transfected with indicated plasmids. Experiments were repeated at least three times, with similar results. (C) RT-qPCR analysis of Il6, Ccnd1, Vegfa, Ccl5 and Cxcl12 mRNA levels of Ctrl and Nsd2-KO cells (KPC1199 derived). Experiments were repeated at least three times, with similar results. (D) Normalized read density of Nsd2 and p65 ChIP-seq signals in KPC1199 cells transfected with Nsd2 and p65 plasmids. (E) Snapshot of Nsd2 and p65 ChIP-seq signals in KPC1199 cells transfected with indicated plasmids.

Figure S6.

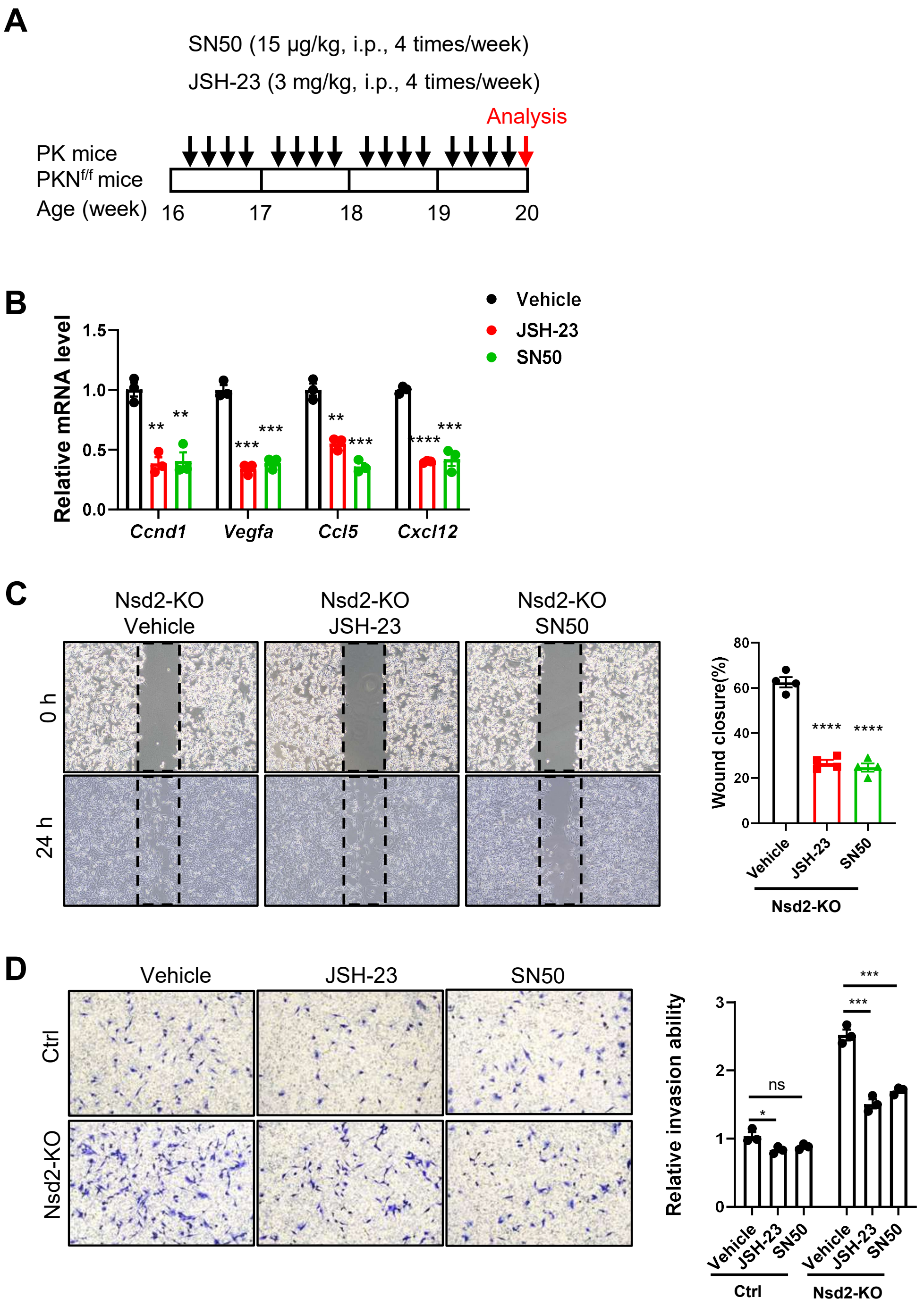

Fig. S6. Inhibition of NF-κB signaling decreased cell migration and invasion of Nsd2-deficient cells. (A) Scheme of treatment given for each injection. (B) RT-qPCR analysis of Ccnd1, Vegfa, Ccl5 and Cxcl12 mRNA levels of PKN$^{f/f}$ mice treated with JSH-23, SN50 or vehicle control. Experiments were repeated at least three times, with similar results. (C) Wound healing assay of Nsd2-KO KPC1199 cells treated with JSH-23, SN50 or vehicle control for 24 hours. Experiments were repeated at least three times, with similar results, and representative images are shown. (D) Transwell-based invasion assay of Ctrl and Nsd2-KO KPC1199 cells treated with JSH-23, SN50 or vehicle control for 24 hours. Experiments were repeated at least three times, with similar results, and representative images are shown.

Figure S7.

A

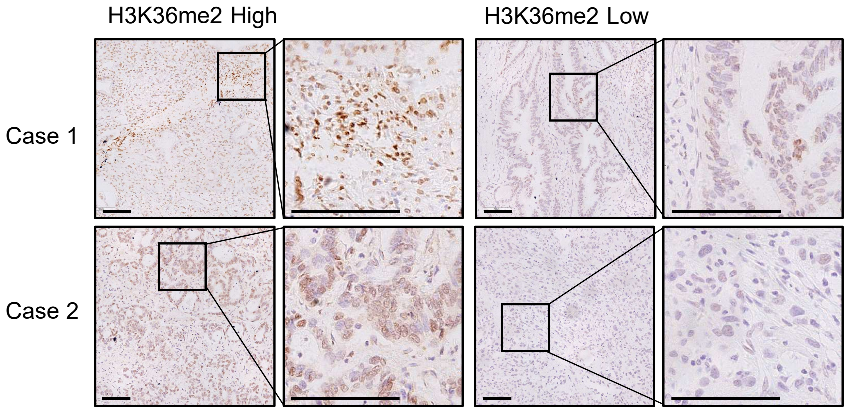

B

	H3K36me2	
	**High**	**Low**
**Normal**	18	28
**PDAC**	30	59
	χ^2=0.7813	*P*=0.3768

C

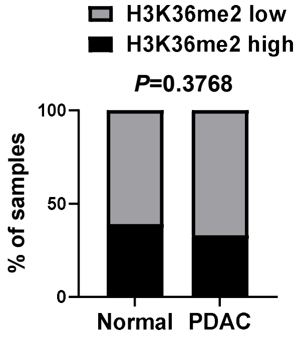

Fig. S7. **H3K36me2 expression in PDAC patients.**

(A) Representative IHC staining indicates high and low level of H3K36me2 in PDAC tissue array. Scale bars: 100 μm. (B) Statistics of high and low level of H3K36me2 in pericarcinomatous samples and PDAC samples. (C) H3K36me2 level in pericarcinomatous samples and PDAC samples is quantified (χ2 test). The data represent the mean ± S.E.M., and statistical significance was determined by a two-tailed Student's t-test unless otherwise indicated.

Figure S8.

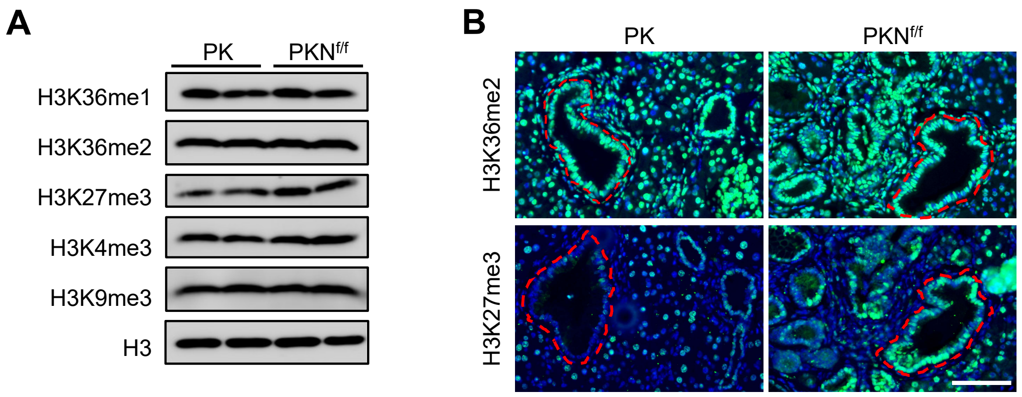

Fig. S8. The level of H3K27me3 was significantly increased in PKN$^{f/f}$ mice.

(A) Western blotting analysis of H3K36me1, H3K36me2, H3K27me3, H3K4me3, H3K9me3 and H3 in pancreas tissues of PK and PKN$^{f/f}$ mice. Experiments were repeated at least three times, with similar results. (B) Immunofluorescence analysis of H3K36me2 and H3K27me3 in pancreatic tissues from indicated mice (n = 5 per group). Experiments were repeated at least three times, with similar results, and representative images are shown. Scale bars: 50 μm.
